# Evaluation of Road Transport Pollutant Emissions from Transporting Building Materials to the Construction Site by Replacing Old Vehicles

**DOI:** 10.3390/ijerph17249316

**Published:** 2020-12-13

**Authors:** Sehee Han, Seunguk Na, Nam-Gi Lim

**Affiliations:** 1 Department of Architectural Engineering, College of Engineering, Dankook University, 152 Jukjeon-ro, Suji-gu, Yongin-si 16890, Gyeonggi-do, Korea; edu.hansh@gmail.com; 2 Department of Architectural Engineering, College of Architecture and Design, Tongmyoung University, 428 Sinseon-ro, Nam-gu, Busan 48520, Korea; ing@tu.ac.kr

**Keywords:** road pollutant emissions, construction industry, diesel vehicles, nitrous oxide, volatile organic compounds, particulate matter, carbon monoxide, transportation

## Abstract

Since the life cycle of a building spans more than 50 years, studies of the environmental impacts in the construction industry have focused on reducing the energy consumption and greenhouse gas emissions during the operation and maintenance phase. The products of the construction industry are assembled using various building materials manufactured outside of the construction site. Consequently, it is essential that the manufactured building materials be transported to the construction site using various types of transportation methods. However, there is a lack of studies that assess the pollutant emissions of road transport while executing a construction project. The purpose of this study is to investigate the changes in the road pollutant emissions when the old diesel vehicles for transporting building materials are replaced according to enhanced pollutant emission regulations. In this study, we found that approximately 89, 64, 77, and 64% of NO_x_, VOC, PM, and CO, respectively, were emitted during transportation of building materials as a proportion of the emissions during the construction of the structure. The analyzed results also show that about 10, 35, 23, and 35% of NO_x_, VOC, PM, and CO, respectively, were generated from material transportation as a proportion of the emissions from finishing the work. It is expected that a reduction in pollutant emissions from transporting building materials of up to approximately 64, 39, 49, and 27% of NOx, VOC, PM, and CO, respectively, can be achieved when vehicles registered before 2003 are replaced with ones that adhere to the tightened regulations.

## 1. Introduction

The construction industry is regarded as one of the main contributors to environmental damage since it consumes a large volume of energy and emits various pollutants [1,2,3]. This industry accounts for about 40% of global energy consumption and approximately 35% of total greenhouse gas emissions [4,5,6]. In particular, the life cycle of a building spans over a period of more than 50 years, which is much longer than the lifespans of other products or services [7,8,9]. Due to the long life cycles of buildings, the majority of studies regarding energy consumption and reducing the environmental impacts of buildings have almost exclusively focused on energy consumption during the operation and maintenance phase while ignoring other stages in the life cycles of buildings [3,6,10,11]. Moreover, the operation and maintenance phase of a building uniquely consumes the majority of energy and greenhouse gas emissions. As a result, one of the main approaches to mitigate environmental impacts in the construction industry is to lower energy consumption in the operation and maintenance stage [10,11,12]. Operating energy consists of the energy used for heating, cooling, ventilation, appliances, and other auxiliary systems by the occupants of a building. One of the most prevalent approaches to lowering the operating energy is the zero-energy building concept, which maximizes the efficiency of energy consumption during the operation and maintenance of a building [10,13,14]. As the energy consumption and environmental impacts during the operation and maintenance phases decrease with the application of newly developed technologies, the environmental impacts that occur during the pre-operational stages, such as the manufacturing of materials, transportation, and construction, increase gradually as a proportion of the whole.

Several studies indicate that the main contributor to environmental damage during the pre-operational stage of a building project is the manufacturing of building materials [15,16,17,18]. A number of studies have claimed that reducing the environmental burden of manufacturing building materials would be an effective way to manage environmental damage during the pre-operational stage. González and Navarro [19], for example, argue that selecting materials with low environmental impacts during the design stage would make it possible to reduce the carbon dioxide emissions from that construction site by up to 28%. Similarly, it was stated that the application of low-carbon materials would make it possible for Dutch residential houses to reduce approximately 50% of their carbon dioxide emissions [20]. Moreover, a few academics noted that replacing high-strength building materials would be an effective approach to mitigate environmental impacts during the construction stage of a building. [2,21,22,23]. For example, Cho and Na [21] indicated that the application of high-strength reinforcing bars would make it possible to reduce energy consumption during manufacturing, as well as lower the amount of rebar used for the construction of high rise buildings. In this study, high-strength building materials made it possible to reduce the quantities of building materials for the optimal design of a building.

As mentioned above, most studies regarding the environmental impact during the pre-operational phases of buildings mainly deal with reductions in the environmental load in the manufacturing of building materials [24,25,26,27]. On the other hand, there has been little focus on the environmental burden that comes from the transportation of building materials to construction sites. While a few studies have examined the environmental impacts during the construction stage of buildings, the impact of the transportation stage can be relatively smaller than any other phases of a construction project [6,12,24]. Although the proportion of the environmental impact from this phase is negligible, the transportation stage has a significant role in executing construction projects. Unlike other industrial items manufactured in factories, several building materials produced outside of the construction site are delivered to the construction site and assembled on-site. Consequently, it is essential that the manufactured building materials be transported to the construction site using various types of transportation methods. In addition, the frequency of construction works in urban areas where population densities are much higher than regional averages has increased in recent years. Therefore, the contribution of pollutants from motor vehicles to the exposure risk of the surrounding residents is much higher, even though the environmental impact from the transportation of building materials accounts for only a small proportion compared to the other phases of the whole life cycle.

Most of the vehicles that transport building materials use diesel-powered engines, which have been highlighted as one of the main sources of road pollutants, such as nitrous oxide and particulate matter. For this reason, regulations on diesel-powered vehicles have been enhanced in many countries, such as the United Kingdom, Germany, and South Korea. However, it was found that approximately 28% of commercial vehicles in South Korea were registered before 2002 when the regulations on air pollutant emissions were weaker than current ones [28,29,30]. Since the Volkswagen emissions scandal in 2015, the regulations on diesel-powered engines have been strengthened worldwide, and the vehicles used in the construction industry, such as ready-mixed concrete mixers and heavy goods vehicles, are no exception to these new rules. However, there is a lack of studies that assess the pollutant emissions of road transportation after replacing unregulated old vehicles from the perspective of the construction industry. The purpose of this study is to investigate the changes in the road pollutant emissions when the old diesel vehicles for transporting building materials are replaced with vehicles that follow the enhanced pollutant emission regulations. The pollutants in this study refer to nitro oxides (NO_x_), volatile organic compounds (VOC), particulate matter (PM), and carbon monoxide (CO). This paper consists of six sections, as described below. After the introduction section, the relevant literature is reviewed in Section 2. In Section 3, the research methodology is described, and the results and a discussion are presented in Section 4. In the Section 5., the conclusions and implications of the study are presented.

## 2. Literature Review

The pollutant emissions of road transportation have aroused strong interest in many countries and many disciplines. These studies mainly focused on the evaluation of vehicular pollutant emissions and the emission factors under different national backgrounds. The studies in this context are based on the estimation standards from the 2006 IPCC (Intergovernmental Panel on Climate Change) Guidelines [31]. In the estimation of pollutant emissions, it is important to enhance the precision of the relevant information, such as the fuel consumption of each type of vehicle and the coefficient of pollutants. To obtain more precise information, various studies have sought to measure different kinds of vehicles, scenarios, and conditions [32]. The United States and some of the member countries of the European Union have estimated the pollutant emissions from the transportation sector to measure the pollutant emission factors and update the national vehicle emission inventories [33,34,35].

Moreover, a number of studies have tried not only to investigate the characteristics of the pollutant emissions of road transportation but also to examine the mitigation potential of these vehicular emissions. For example, Li et al. [32] carried out a potential reduction of greenhouse gas and pollutant emissions of road transportation by adopting an inventory calculation and scenario analysis. The authors suggested that road freight transportation would play a key role in mitigating the reduction of air emissions by about 85–92% compared to the 2012 values. Moreover, various researchers have examined the reduction of vehicular pollutant emissions and road conditions, such as traffic signal timing, the speed limits of roads, etc. Madireddy et al. [36] investigated the reduction of vehicle emissions and traffic management measures, focusing on speed limit reductions and traffic light control. In a similar vein, Li et al. [37] investigated the environmental impacts of traffic signal timing in the Chinese road context. In particular, the majority of studies have claimed that the policy implications related to the reduction of pollutant emissions from road transportation represent one of the key factors to mitigate adverse consequences.

Although many studies have analyzed the pollutant emissions of road transportation, there has been little research on pollutant emissions from transporting building materials in the construction industry. While Paik and Na [4], for example, dealt with the estimation of carbon dioxide emissions from a construction project, the CO_2_ emissions from transportation were far smaller than those of other phases, even though they were of significance for the building construction. Similarly, Sandanayake et al. [38] conducted a process-based greenhouse gas emission quantification study that showed about 14% of greenhouse gas emissions to be caused by transportation in foundation construction projects. Moreover, several academics have quantified the carbon dioxide levels of building material transportation, rather than the pollutant emissions of road transportation. da Costa and da Costa [39] estimated the CO_2_ emissions from transporting building materials to the construction site in the Brazilian context. In addition, the authors suggested a new scenario to reduce the carbon dioxide emissions of road transportation by adopting a combination of an existing road system with rail for long-distance transportation. However, there is a lack of studies focusing on the different relevant scenarios, such as the substitution of old vehicles, different speeds, traffic conditions, etc. In this paper, we comprehensively examine the road pollutant emissions during the transport of building materials, which can help to mitigate the environmental impacts from the pollutant emissions when transporting building materials during the construction phase of a building.

## 3. Research Methods

This study analyses the changes in road transport pollutant emissions from transporting building materials after replacing the old vehicles registered before 2003 with transport methods that meet the current air pollutant emissions standards. The number of registered commercial vehicles used in this study was based on vehicle registration data from the Korean Statistical Information Service [40] and the National Logistics Information Centre in 2017 [41]. In addition, the road pollutants generated from transporting building materials that we investigated were limited to nitro oxides (NO_x_), volatile organic compounds (VOC), particulate matter (PM), and carbon monoxide (CO). To achieve the goals of this study, the calculation of road transport pollutant emissions was divided into four steps as follows: (1) selecting which cases in which to evaluate the road pollutant emissions; (2) quantity surveying of the studied building; (3) classifying the types of vehicles used for each building material and calculating the number of vehicles; and (4) evaluating the road pollutant emissions of each building material.

A practical case of a commercial building in South Korea, the construction of which started in 2017 and was completed in 2019, was selected to evaluate the road pollutant emissions from transporting building materials. This case was also used to investigate the changes from replacing old vehicles with vehicles that meet the enhanced pollutant emission regulations in South Korea. As summarised in Table 1, the case evaluated in this study was located in Gyeonggi-do, South Korea. The studied building has a steel-framed reinforced concrete structure with a 1-storey underground floor and 4 storeys aboveground. The building was designed in compliance with the Structural Concrete Design Code and Commentary of Korea [42]; the minimum design loads and associated criteria for this building and other such structures are defined in (ASCE/SEI 7–10) [43].

**Figure 1:**
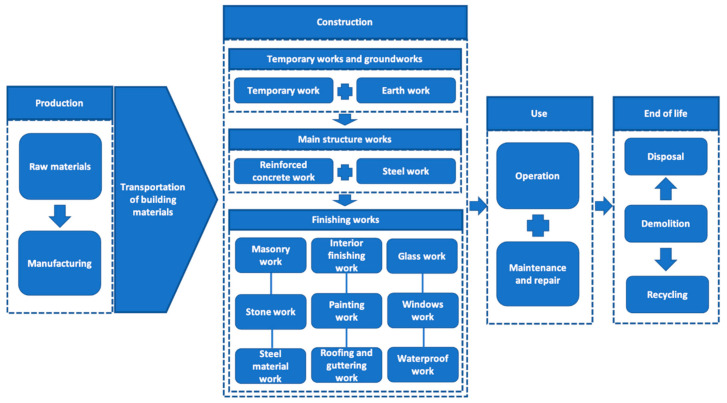
The life cycle of and construction work of a building.

The quantities of the building materials for this construction work were estimated from the bill of quantities. Drawings and the bill of quantities (BOQ) were used to identify the type of construction work and the quantity of building materials used in the studied building. Based on the drawings and BOQ, the construction work was divided into three major categories, which were then sub-divided into 14 sub-tasks in total (See Figure 1). The major types of construction work considered in this study were temporary work, structural work, and finishing work. Since the majority of construction work during foundation work was related to removing soil rather than delivering building materials, the pollutant emissions during this work were excluded. The sub-tasks were temporary work, concrete work, steelwork, masonry work, stonework, interior finishing, waterproof work, roofing and guttering work, steel materials work, window work, glasswork, and painting work. Based on the bill of quantities calculated in the second step, the types and numbers of vehicles for the transportation of each building material were established. Since it was impossible to compute the exact number of old vehicles used for transportation, the ratio of old vehicles used was calibrated based on the number of vehicles registered by year in the registration office.

The pollutant emission factors for each type of commercial vehicle were adopted from the calculation method outlined in the National Air Pollutant Emission Estimation Manual provided by the National Institute of Environmental Research in South Korea [40]. According to the manual [40], the types of commercial vehicles can be divided into categories of less than 5 tons and more than 5 tons. In this study, four different types of transportation (a 1-ton lorry, a 4.5-ton lorry, an 8-ton lorry, and a ready-mixed concrete mixer) were used to convey the building materials from the manufacturers’ location or distribution center to the construction site. In addition, the pollutant emission factors were also classified by year of registration. These factors cover four groups of vehicles: those made before 1995, those made from 1996 to 1997 and 1998 to 2002, and those made after 2003. The pollutant emission factors for each road pollutant evaluated in this study are summarized in Table 2.

Equation (1) was used to determine the emissions of road pollutants from transporting building materials. The elements for computing the road pollutant emissions were the emission factors for each type of vehicle, the transported distance of each building material from the manufacturers’ location to the construction site, and the total number of vehicles for transporting each building material:

(1) $$E_{i,m}=f_{i,t,y}\times K_{m}\times N_{m}$$

where *E_i,m_* are the emissions of the road pollutant *i* (nitrous oxide, volatile organic compounds, particulate matter, or carbon monoxide) from carrying building materials m, *f_i,t,y_* is the emission factor of the road pollutant *i* from transportation method *t* in year *y*, *K_m_* is the travelled distance of the vehicle (in km) with the building material *m*, and *N* is the total number of vehicles used for the transportation of building material *m*.

## 4. Results

### 4.1. Total Road Transport Pollutant Emissions

This section outlines the road transport pollutant emissions from transporting building materials during the construction of a commercial building. In this study, the major construction work involved in a commercial building was divided into temporary work, structural work, and finishing work. In addition, these three major types of construction work were further broken down into 14 types of sub-tasks. Temporary work is composed of only one sub-task, temporary work; structural work is divided into two sub-tasks, concrete work and steelwork; finishing work consists of 11 types of sub-tasks: masonry work, stonework, tile work, interior finishing, waterproofing, roofing and guttering, steel material work, plastering work, window work, glasswork, and painting work.

#### 4.1.1. Total Road Transport Pollutant Emissions from Construction Work

Table 3 outlines the road transport pollutant emission results from transporting building materials based on the number of vehicles registered in 2016. The proportion of pollutant emissions from transporting building materials by construction type shows a similar trend for all road transport pollutants. It was found that most road transport pollutants from transporting building materials were released during structural work. As shown in Table 3, the assessment results show that the transport of building materials for structural work contributed more than 89%, 64%, 77%, and 64% of the overall value of NO_x_, VOC, PM, and CO, respectively, and that the absolute value of each pollutant was approximately 4.620, 0.088, 0.062, and 0.35 ton for NO_x_, VOC, PM, and CO, respectively (see Table 3). In addition, the test results show that 0.519 tons of NOx, 0.463 tons of VOC, 0.181 tons of PM, and 1.89 tons of CO were released while transporting building materials for finishing work. The NO_x_ emitted from transporting building materials for finishing work accounts for about 10% of the total pollutant emissions. For VOC, PM, and CO emissions accounting for approximately 34% of the total emissions, were identified from the transport of materials used for finishing work. As indicated in the results of this study, the building materials used for structural work accounted for the largest proportion of emissions, even though the number of building materials was less than that for finishing work. This seems to be because the quantity of building materials used for structural work was significantly higher than that for finishing work.

The emissions of road transport pollutants from transporting building materials for each sub-task are summarised in Table 4. In the case of structural work, which emits the most road transport pollutants, most road transport pollutant emissions occurred when transporting ready-mixed concrete, steel decking, and steel forms. It was found that the proportion of the total amount of road transport pollutants released by ready-mixed concrete was approximately 67% for NO_x_, VOC, PM, and CO. Additionally, about 25% and 6% of the road transport pollutants were emitted while transporting steel decking and steel forms, respectively. In particular, even though steel decking was made of galvanized steel, which is relatively lighter than steel, the number of vehicles needed for transportation was higher since the unit size of steel decking is very large. For this reason, the amount of road transport pollutant emissions from steel decking is high, even though the bill of quantities for steel decking was smaller than that for other building materials in the structural work. In addition, the unit size of steel forms is smaller than that of plywood forms, but the number of lorries needed for transporting, as well as the amount of pollutant emissions, were larger for steel forms.

For finishing work, the proportion of total road transport pollutant emissions was lower than that for structural work, despite the number of building materials required being higher. In this study, the emissions of NO_x_ from transporting building materials for finishing work accounted for about 10% of the total. Furthermore, it was determined that approximately 23%, 34%, and 35% of PM, VOC, and CO, respectively, were produced during the transportation of building materials for finishing work. There are several interesting aspects related to the transportation of building materials for finishing work. For stonework, the amount of granite required in the construction project was found to be lower than that for other building materials. However, due to the capacity of the loading machine and the unit weight of each piece of granite, the number of lorries needed to transport this material was quite high, and these vehicles emitted large volumes of pollutants into the air. Furthermore, the transportation of glass also required a large number of lorries since glass is brittle and needs to be transported with great care. When transporting glass to the construction site, it is impossible to carry glass that is tightly stacked like other materials, such as gypsum boards or insulators. To prevent breaking the glass during transportation, many lorries with a low number of glass sheets are required. Based on these results, we conclude that the emissions of pollutants from transporting building materials by road were dominated by each element’s unit weight, rather than the size of the building material being transported.

#### 4.1.2. Road Pollutant Emissions by Building Material

Table 5 shows the results for how each major building material contributed to the pollutant emissions during road transportation. The results indicate that three major building materials caused more than 80% of the total NO_x_ emissions during the transport of ready-mixed concrete, steel decking, and rolled steel sheets. In the case of VOC, PM, and CO, the road pollutant emissions from transporting ready-mixed concrete, steel decking, and rolled steel sheets showed similar results to the results for NO_x_ and accounted for about 55, 65, and 54%, respectively. In particular, the transportation of ready-mixed concrete contributed to approximately 51, 36, 43, and 35% of the pollutant emissions for NO_x_, VOC, PM, and CO, respectively. Since the structural system of the studied building was a steel-framed reinforced concrete structure, ready-mixed concrete, steel decking, and rolled steel sheets contributed the majority of emissions of NO_x_, VOC, PM, and CO during their transportation. Particularly, steel decking and rolled steel sheets required more vehicles because the unit size of each is relatively large compared to that of other building materials of similar weight. In this study, the unit size of each piece of building material was one of the determining factors for allocating the number of vehicles needed for transporting that material. Even though the weight of each unit may be smaller, e.g., for gypsum boards, the required number of vehicles was greater than that for other building materials.

While transporting building materials for steel construction work, most of the pollutants were emitted during the transportation of rolled steel sheets. In the case of NO_x_, approximately 73% of the total road transportation pollutants were emitted while transporting rolled steel sheets. Similarly, about 64%, 69%, and 63% of the VOC, PM, and CO, respectively, were released while transporting this material (see Table 5). In addition, the emissions of pollutants during the transportation of building materials used for connecting the steel members and fireproofing paint accounted for about 1.6%, 10%, 6%, and 11% of NO_x_, VOC, PM, and CO, respectively. In the case of building materials for steel construction work, both the weight and volume were larger than those for other building materials in this study. In addition, it was impossible to convey building materials that exceeded 12 m in length due to the regulations set out in the Road Traffic Act in South Korea. Along with these factors, the steel materials had a longer transport distance than that for other building materials because the distribution centers for these items are not near central district areas but are instead directly shipped from the manufacturing location. Due to these reasons, the amount of road transport pollutant emissions was higher for steel. To mitigate the emissions of road pollutants during the transportation of steel, we should not only minimize the lead time between sub-tasks but also reallocate the transportation time to avoid road congestion.

### 4.2. Changes in Road Transport Pollutant Emissions by Replacing Old Vehicles

In this section, we investigate how road transport pollutant emissions will change when the old vehicles are replaced by new ones registered after 2003, when the regulations on pollutant emissions for vehicles were tightened. The changes in pollutant emissions were examined for a case where 10% of old vehicles were replaced by new vehicles. The changes in road pollutant emissions were considered according to the type of construction work and the building material.

**Figure 2:**
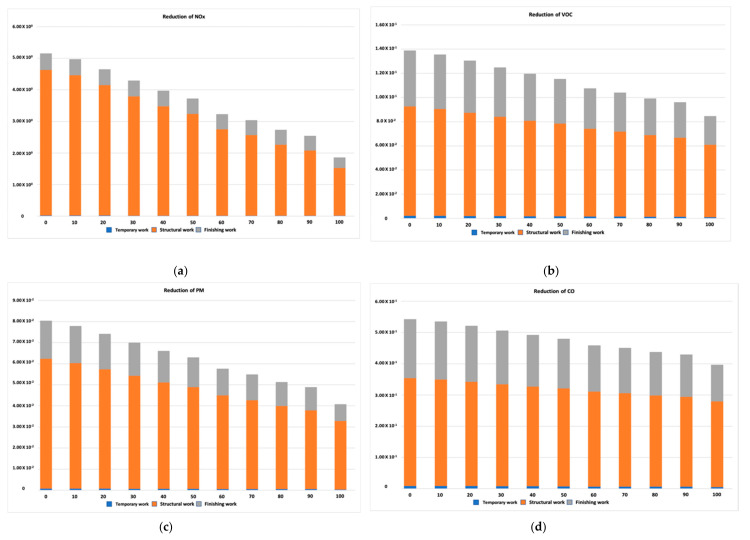
Reduction of the various road pollutants. (**a**) Reduction of NO_x_, (**b**) reduction of VOC, (**c**) reduction of PM, (**d**) Reduction of CO.

Figure 2 shows the variation in pollutant emissions for the four road pollutants released while transporting building materials as the number of old vehicles was lowered. As indicated in Figure 2, the amount of road transport pollutant emissions tended to decrease as the proportion of old vehicles registered before 2002 decreased. When a reduction in the total road pollutant emissions was considered, all four pollutants showed a decreasing tendency as the old vehicles were replaced with vehicles that adhere to the current pollutant emissions reduction regulations. These results suggest that the total pollutant emissions from transporting building materials by road would be reduced by up to 64, 39, 49, and 27% of NO_x_, VOC, PM, and CO, respectively, if all the unregulated vehicles were replaced by enhanced ones (see Table 6). In addition, the replacement of old vehicles for transporting building materials in a construction project would make it possible to not only directly reduce the amount of particulate matter but also to mitigate the emissions of nitrogen oxide, which is one of the key factors in the generation of particulate matter. In particular, it was observed that the reduction in the ratio of NO_x_ emitted was significant in lowering the total emissions.

Moreover, the results of this study show that the characteristics of how pollutant emissions were reduced are different depending upon the type of pollutant source. The emission characteristics of NO_x_ showed a distinct difference according to the age of the vehicle, while other factors, such as VOC, PM, and CO, only showed slight differences according to the ages of the vehicles. Based on these results, it is expected that replacing old vehicles would contribute to reducing the particulate matter emitted over the course of a construction project.

Along with a reduction of the total pollutant emissions during the transportation of building materials by road, the pollutant emission reductions from the three major types of construction work are shown in Table 7. For temporary work, NO_x_, VOC, PM, and CO were reduced by approximately 18, 49, 56, and 38%, respectively, as all of the old vehicles were replaced with new ones that adhere to the current pollutant emissions regulations. The structural work shows the largest pollutant emissions reduction among the three major kinds of construction work. In particular, a 67% reduction of NO_x_ in structural work is achieved with new vehicles compared to the results of the pollutant emissions based on the data from commercial vehicle registrations in 2016. Moreover, a 34, 47, and 21% reduction in VOC, PM, and CO, respectively, was observed from transporting building materials for structural work with newer vehicles.

While the finishing work examined in this study required more different building materials compared to the other types of major construction work, the quantities of the items were smaller than those in the other work. In other words, the road transport pollutant emissions from building materials for finishing work were relatively higher than those from other work, even though the quantities for this type of work were smaller than those for temporary and structural work. This seems to be the nature of road transportation, where vehicles travel even when they are not fully loaded. While the quantity of ceramic tiles in tile work, for example, might be 600 kg, the transportation method for this item might involve a 1-ton lorry, so the emissions must be calculated on a per vehicle basis, even though the payload is only 60% full capacity.

### 4.3. Discussion

In this section, several factors, such as the significance, usefulness, and limitations of this study, along with further research directions, are presented. As the measures for reducing particulate matter and the health and safety measures for workers on-site increase, there is growing interest in reducing pollutant emissions in various fields, including the construction industry. In particular, one of the main sources of pollutant emissions during construction projects is transporting building materials from the manufacturer’s location or distribution centers to the construction site.

The findings of this research show the pollutant emissions created by transporting building materials to a construction site, as well as the potential benefits of replacing old vehicles. Previous studies on the environmental impact of construction projects have focused on verifying the carbon contained in the building materials, as well as on providing suggestions of new technologies for reducing the environmental burden during the operational and maintenance stages of a building. Moreover, a number of studies have maintained that the environmental impact from the transportation of building materials is negligible, so the impacts of these emissions have been less well studied compared to the impacts of emissions during other stages in the life cycle of a building. However, this research tried not only to calculate the emissions of pollutants from the transportation of building materials but also to analyze the possibilities for pollutant reductions during the transportation stage of a building’s life cycle. It is possible for the findings in this research to be used as a basis for evaluating a reduction in pollutant emissions while transporting building materials after replacing old vehicles for various types of building projects.

Although the findings of this study are beneficial for evaluating the potential reduction of road pollutant emissions while transporting building materials in a construction project, there are several points that could improve the results and usefulness of this work. The pollutant emissions produced during building material transportation examined in this study were only based on one office building. To further confirm the potential reductions in pollutant emissions by replacing old vehicles in construction projects, further research could consider other types and purposes of building projects, such as residential and commercial buildings. Since this study only focused on one type of building, the applicability of the results is limited. Therefore, further studies should be carried out to analyze major building methods and materials in different construction projects. In addition, the results of this study were based on only one structural system—a steel-framed reinforced concrete structure. However, the results for the reductions in road pollutant emissions from construction projects would be more accurate and widely applicable if different structural systems with different building materials and types of construction work were considered. Through an analysis of the road pollutant emissions from different types of buildings with different structural systems, the optimal location of manufacturing and the optimal number of vehicles for transportation could be determined in further research. Moreover, the emission factor for each pollutant in this study was determined based on the National Air Pollutant Emissions Method Manual. However, to enhance the accuracy of the emission factors used and the results, real road testing, and evaluations of the emissions for each type of building material transportation method should be conducted in the future.

## 5. Conclusions

The goal of this study was to examine the changes in vehicular pollutant emissions while transporting building materials by road when the old vehicles were replaced with newer ones that meet the enhanced pollutant emission regulations. Based on bills of quantities and drawings, three major types of construction work were identified (i.e., temporary, structural, and finishing work). In addition, the major construction works were divided into the 35 types of building materials required to be transported from the manufacturers’ location to the construction site. To calculate the pollutant emissions of road transport, the emission factors of the pollutants were adopted from the National Air Pollutant Estimation Manual.

In this study, it was found that approximately 89, 64, 77, and 64% of the total NO_x_, VOC, PM, and CO were emitted while transporting building materials for structural work. These results also showed that about 10, 35, 23, and 35% of the total NO_x_, VOC, PM, and CO were generated during material transportation for finishing work. Furthermore, pollutant emissions were analyzed by building material, and the results showed that ready-mixed concrete, steel decking, and rolled steel sheets were the three primary materials that contributed to the emissions of pollutants during the transportation of building materials. In this study, the unit size of each type of building material was found to be a significant factor in determining the number of vehicles. Even though some of the building materials are quite lightweight, such as gypsum boards and rubber insulators, the number of lorries needed for transporting those materials was higher because the unit size of these materials was larger than that of others.

We also investigated the change in road transport pollutant emissions as a certain number of old vehicles were replaced with new ones that met the tightened pollutant emission regulations. These results indicate that all four pollutant types were lowered as the old vehicles were substituted with newer commercial vehicles that adhered to the tightened regulations. In this study, our calculations showed that up to approximately a 64, 39, 49, and 27% reduction in the respective NO_x_, VOC, PM, and CO emissions from transporting building materials could be achieved if vehicles registered before 2003 were replaced by newer ones that comply with the tightened regulations.

The results of this research could be applied in future studies on road-pollutant emissions from various types of different buildings, such as residential buildings, offices, etc. Although we observed potential benefits from reducing the environmental burden by replacing old vehicles, further research that considers various transportation methods should be carried out to better understand the context of the results in this study.

## Figures and Tables

**Table 1:** The profile of the building.

Description	Content
Location of the building	Gyeonggi-do, Suwon, South Korea
Local district	A 1st tier residential area
Scale of the building	4 storeys aboveground and 1 storey underground
Structural system	Steel-framed reinforced concrete structure
Type of the building	Commercial building
Land area	26,240.54 m^2^
Total floor area	12,923.84 m^2^ (1st floor: 2935.32 m^2^, 2nd floor: 2685.47 m^2^, 3rd floor: 2697.27 m^2^, 4th floor: 2144.25 m^2^, and underground floor: 2461.53 m^2^)

**Table 2:** The emission factors for each type of commercial vehicles (unit: g/km [40]).

Year	Classification	NOx	VOC	PM	CO
Before 1995	Less than 5 tons	6.36 × 10^0^	1.20 × 10^0^	5.54 × 10^−1^	4.54 × 10^0^
	5 tons or more	4.16 × 10^2^	2.10 × 10^0^	1.79 × 10^0^	5.99 × 10^0^
1996–1997	Less than 5 tons	5.76 × 10^0^	9.33 × 10^−1^	4.47 × 10^−1^	3.78 × 10^0^
	5 tons or more	2.34 × 10^1^	1.35 × 10^0^	1.21 × 10^0^	5.47 × 10^0^
1998–2002	Less than 5 tons	4.70 × 10^0^	8.86 × 10^−1^	2.71 × 10^−1^	2.84 × 10^0^
	5 tons or more	2.12 × 10^1^	1.33 × 10^0^	1.07 × 10^0^	3.55 × 10^0^
After 2003	Less than 5 tons	3.12 × 10^0^	2.31 × 10^−1^	7.43 × 10^−2^	1.15 × 10^0^
	5 tons or more	1.50 × 10^1^	5.78 × 10^−1^	3.23 × 10^−1^	2.63 × 10^0^

**Table 3:** The total pollutant emissions based on the number of vehicles registered in 2016.

Type of Work	Emissions (ton)				Proportion (%)			
	NO_x_	VOC	PM	CO	NO_x_	VOC	PM	CO
Temporary	1.78 × 10^−2^	2.11 × 10^−3^	7.83 × 10^−4^	8.68 × 10^−3^	0.3	1.6	1.0	1.6
Structure	4.62 × 10^0^	8.76 × 10^−2^	6.16 × 10^−2^	3.45 × 10^−1^	89.6	64.4	76.6	63.5
Finishing	5.19 × 10^−1^	4.63 × 10^−2^	1.81 × 10^−2^	1.89 × 10^−1^	10.1	34.0	22.5	34.9
Total	5.15 × 10^0^	1.36 × 10^−1^	8.04 × 10^−2^	5.43 × 10^−1^	100	100	100	100

**Table 4:** Total pollutant emissions of sub-tasks.

Type of Work	Emissions (ton)				Proportion (%)			
	NO_x_	VOC	PM	CO	NO_x_	VOC	PM	CO
Temporary work	1.78 × 10^−2^	2.11 × 10^−3^	7.83 × 10^−4^	8.68 × 10^−3^	0.35	1.52	0.97	1.60
Concrete work	3.87 × 10^0^	7.67 × 10^−2^	5.23 × 10^−2^	2.93 × 10^−1^	75.05	55.22	65.01	53.95
Steel work	7.50 × 10^−1^	1.38 × 10^−2^	9.31 × 10^−3^	5.21 × 10^−2^	14.55	9.94	11.57	9.59
Masonry work	1.28 × 10^−2^	1.52 × 10^−3^	5.68 × 10^−4^	6.27 × 10^−3^	0.25	1.09	0.71	1.15
Stonework	1.51 × 10^−1^	2.27 × 10^−3^	1.63 × 10^−3^	8.47 × 10^−3^	2.93	1.63	2.03	1.56
Tile work	1.45 × 10^−2^	1.79 × 10^−3^	6.74 × 10^−4^	7.28 × 10^−3^	0.29	1.29	0.84	1.34
Interior finishing work	1.85 × 10^−1^	2.19 × 10^−2^	8.15 × 10^−3^	9.03 × 10^−2^	3.59	15.77	10.13	16.63
Waterproofing work	1.00 × 10^−2^	1.23 × 10^−3^	4.65 × 10^−4^	5.03 × 10^−3^	0.19	0.89	0.59	0.93
Roofing and guttering work	2.10 × 10^−2^	2.60 × 10^−3^	9.78 × 10^−4^	1.06 × 10^−2^	0.41	1.87	1.22	1.95
Steel materials work	6.00 × 10^−3^	7.22 × 10^−4^	2.71 × 10^−4^	2.97 × 10^−3^	0.12	0.52	0.34	0.55
Plastering work	1.01 × 10^−2^	1.23 × 10^−3^	4.68 × 10^−4^	5.04 × 10^−3^	0.20	0.89	0.59	0.93
Window work	1.63 × 10^−2^	1.97 × 10^−3^	7.25 × 10^−4^	8.00 × 10^−3^	0.32	1.42	0.90	1.47
Glass work	4.34 × 10^−2^	5.17 × 10^−3^	1.93 × 10^−3^	2.13 × 10^−2^	0.84	3.72	2.40	3.92
Painting work	4.85 × 10^−2^	5.89 × 10^−3^	2.20 × 10^−3^	2.41 × 10^−2^	0.94	4.24	2.73	4.44

**Table 5:** Total pollutant emissions by building material.

Building Materials	Emissions (ton)				Proportion (%)			
	NO_x_	VOC	PM	CO	NO_x_	VOC	PM	CO
Batter boards	3.08 × 10^−3^	3.71 × 10^−4^	1.37 × 10^−4^	1.51 × 10^−3^	0.06	0.27	0.17	0.28
Steel support	1.47 × 10^−2^	1.74 × 10^−3^	6.46 × 10^−4^	7.17 × 10^−3^	0.29	1.25	0.80	1.32
RMC	2.61 × 10^0^	4.93 × 10^−2^	3.47 × 10^−2^	1.88 × 10^−1^	50.64	35.48	43.15	34.58
Rebars	3.98 × 10^−2^	4.69 × 10^−3^	1.74 × 10^−3^	1.93 × 10^−2^	0.77	3.38	2.16	3.55
Forms (Plywood)	6.14 × 10^−3^	7.48 × 10^−4^	2.82 × 10^−4^	3.06 × 10^−3^	0.12	0.54	0.35	0.56
Forms (Steel)	2.37 × 10^−1^	4.39 × 10^−3^	3.12 × 10^−3^	1.67 × 10^−2^	4.60	3.16	3.88	3.07
Steel decking	9.74 × 10^−1^	1.76 × 10^−2^	1.24 × 10^−2^	6.60 × 10^−2^	18.90	12.67	15.42	12.14
H beams	7.26 × 10^−2^	1.20 × 10^−3^	8.64 × 10^−4^	4.44 × 10^−3^	1.41	0.86	1.07	0.82
Rolled steel sheets	5.47 × 10^−1^	8.84 × 10^−3^	6.38 × 10^−3^	3.30 × 10^−2^	10.61	6.36	7.93	6.07
Square steel tubes	1.15 × 10^−1^	1.82 × 10^−3^	1.33 × 10^−3^	6.64 × 10^−3^	2.23	1.31	1.65	1.22
Angles	2.13 × 10^−3^	2.80 × 10^−4^	1.05 × 10^−4^	1.11 × 10^−3^	0.04	0.20	0.13	0.20
Anchoring bolts	8.12 × 10^−4^	1.15 × 10^−4^	4.60 × 10^−5^	4.53 × 10^−4^	0.02	0.08	0.06	0.08
High tension bolts	9.43 × 10^−4^	1.34 × 10^−4^	5.35 × 10^−5^	5.26 × 10^−4^	0.02	0.10	0.07	0.10
Stud bolts	3.29 × 10^−3^	3.91 × 10^−4^	1.46 × 10^−4^	1.61 × 10^−3^	0.06	0.28	0.18	0.30
Fireproof painting	8.97 × 10^−3^	1.06 × 10^−3^	3.87 × 10^−4^	4.34 × 10^−3^	0.17	0.76	0.48	0.80
Bricks	1.28 × 10^−2^	1.52 × 10^−3^	5.68 × 10^−4^	6.27 × 10^−3^	0.25	1.09	0.71	1.15
Granite	1.51 × 10^−1^	2.27 × 10^−3^	1.65 × 10^−3^	8.47 × 10^−3^	2.93	1.63	2.05	1.56
Ceramic tiles	6.39 × 10^−3^	8.04 × 10^−4^	3.05 × 10^−4^	3.25 × 10^−3^	0.12	0.58	0.38	0.60
Porcelain tiles	8.11 × 10^−3^	9.85 × 10^−4^	3.69 × 10^−4^	4.03 × 10^−3^	0.16	0.71	0.46	0.74
Gypsum boards	3.05 × 10^−2^	3.57 × 10^−3^	1.32 × 10^−3^	1.48 × 10^−2^	0.59	2.57	1.64	2.72
Rubber boards	3.44 × 10^−2^	4.11 × 10^−3^	1.53 × 10^−3^	1.69 × 10^−2^	0.67	2.96	1.90	3.11
Insulators	2.18 × 10^−2^	2.61 × 10^−3^	9.73 × 10^−4^	1.07 × 10^−2^	0.42	1.88	1.21	1.97
Sound absorbing materials	4.32 × 10^−2^	5.13 × 10^−3^	1.92 × 10^−3^	2.12 × 10^−2^	0.84	3.69	2.39	3.90
Light-weight partition walls	5.50 × 10^−2^	6.49 × 10^−3^	2.41 × 10^−3^	2.68 × 10^−2^	1.07	4.67	3.00	4.93
Waterproof liquid	5.30 × 10^−3^	6.37 × 10^−4^	2.39 × 10^−4^	2.62 × 10^−3^	0.10	0.46	0.30	0.48
Liquid rubber waterproof sealant	4.74 × 10^−3^	5.95 × 10^−4^	2.26 × 10^−4^	2.41 × 10^−3^	0.09	0.43	0.28	0.44
Stainless pipes	1.76 × 10^−3^	2.18 × 10^−3^	8.25 × 10^−4^	8.88 × 10^−3^	0.34	1.57	1.03	1.63
Roof drains	3.35 × 10^−3^	4.14 × 10^−4^	1.53 × 10^−4^	1.67 × 10^−3^	0.06	0.30	0.19	0.31
Light-weight frames	3.00 × 10^−3^	3.61 × 10^−4^	1.35 × 10^−4^	1.48 × 10^−3^	0.06	0.26	0.17	0.27
Steel handrails	3.00 × 10^−3^	3.61 × 10^−4^	1.35 × 10^−4^	1.48 × 10^−3^	0.06	0.26	0.17	0.27
Plastering mortar	1.01 × 10^−2^	1.23 × 10^−3^	4.68 × 10^−4^	5.04 × 10^−3^	0.20	0.89	0.58	0.93
Windows frames	8.42 × 10^−3^	1.00 × 10^−3^	3.77 × 10^−4^	4.14 × 10^−3^	0.16	0.72	0.47	0.76
Doors	7.93 × 10^−3^	9.39 × 10^−4^	3.48 × 10^−4^	3.87 × 10^−3^	0.15	0.68	0.43	0.71
Glass	4.34 × 10^−2^	5.17 × 10^−3^	1.93 × 10^−3^	2.13 × 10^−2^	0.84	3.72	2.40	3.92
Paint	4.85 × 10^−2^	5.89 × 10^−3^	2.20 × 10^−3^	2.451 × 10^−2^	0.94	4.24	2.74	4.51

**Table 6:** Reduction of the total pollutant emissions (Unit: %).

	NO_x_	VOC	PM	CO
Temporary work	−17.77	−48.68	−55.57	−37.74
Structural work	−67.34	−33.95	−47.20	−20.61
Finishing work	−35.02	−48.85	−56.10	−37.92
Total reduction ratio	−63.91	−39.14	−49.28	−26.92

**Table 7:** Variations of pollutant emissions for the four road pollutants.

Replacing Ratio		Total Emissions (Unit: ton)										
		0	10	20	30	40	50	60	70	80	90	100
NO_x_	Temporary work	1.78 × 10^−2^	1.78 × 10^−2^	1.76 × 10^−2^	1.70 × 10^−2^	1.69 × 10^−2^	1.68 × 10^−2^	1.60 × 10^−2^	1.60 × 10^−2^	1.58 × 10^−2^	1.58 × 10^−2^	1.46 × 10^−2^
	Structural work	4.62 × 10^0^	4.44 × 10^0^	4.13 × 10^0^	3.77 × 10^0^	3.46 × 10^0^	3.22 × 10^0^	2.74 × 10^0^	2.55 × 10^0^	2.25 × 10^0^	2.06 × 10^0^	1.51 × 10^0^
	Finishing work	5.19 × 10^−1^	5.16 × 10^−1^	5.10 × 10^−1^	5.02 × 10^−1^	4.97 × 10^−1^	4.90 × 10^−1^	4.79 × 10^−1^	4.76 × 10^−1^	4.70 × 10^−1^	4.68 × 10^−1^	3.37 × 10^−1^
	Total	5.15 × 10^0^	4.98 × 10^0^	4.66 × 10^0^	4.29 × 10^0^	3.97 × 10^0^	3.73 × 10^0^	3.23 × 10^0^	3.04 × 10^0^	2.73 × 10^0^	2.55 × 10^0^	1.86 × 10^0^
VOC	Temporary work	2.11 × 10^−3^	2.11 × 10^−3^	2.04 × 10^−3^	1.87 × 10^−3^	1.81 × 10^−3^	1.77 × 10^−3^	1.53 × 10^−3^	1.53 × 10^−3^	1.46 × 10^−3^	1.46 × 10^−3^	1.08 × 10^−3^
	Structural work	9.06 × 10^−2^	8.83 × 10^−2^	8.54 × 10^−2^	8.22 × 10^−2^	7.90 × 10^−2^	7.65 × 10^−2^	7.26 × 10^−2^	7.02 × 10^−2^	6.73 × 10^−2^	6.52 × 10^−2^	5.98 × 10^−2^
	Finishing work	4.63 × 10^−2^	4.52 × 10^−2^	4.31 × 10^−2^	4.07 × 10^−2^	3.89 × 10^−2^	3.70 × 10^−2^	3.35 × 10^−2^	3.24 × 10^−2^	3.04 × 10^−2^	2.95 × 10^−2^	2.37 × 10^−2^
	Total	1.39 × 10^−1^	1.36 × 10^−1^	1.31 × 10^−1^	1.25 × 10^−1^	1.20 × 10^−1^	1.15 × 10^−1^	1.08 × 10^−1^	1.04 × 10^−1^	9.92 × 10^−2^	9.62 × 10^−2^	8.46 × 10^−2^
PM	Temporary work	7.83 × 10^−4^	7.83 × 10^−4^	7.63 × 10^−4^	6.77 × 10^−1^	6.57 × 10^−4^	6.44 × 10^−4^	5.38 × 10^−4^	5.38 × 10^−4^	5.18 × 10^−4^	5.18 × 10^−4^	3.48 × 10^−4^
	Structural work	6.16 × 10^−2^	5.95 × 10^−2^	5.66 × 10^−2^	5.36 × 10^−2^	5.04 × 10^−2^	4.83 × 10^−2^	4.45 × 10^−2^	4.22 × 10^−2^	3.94 × 10^−2^	3.73 × 10^−2^	3.25 × 10^−2^
	Finishing work	1.81 × 10^−2^	1.77 × 10^−2^	1.68 × 10^−2^	1.58 × 10^−2^	1.50 × 10^−2^	1.41 × 10^−2^	1.26 × 10^−2^	1.22 × 10^−2^	1.14 × 10^−2^	1.10 × 10^−2^	7.94 × 10^−3^
	Total	8.04 × 10^−2^	7.79 × 10^−2^	7.41 × 10^−2^	7.00 × 10^−2^	6.61 × 10^−2^	6.30 × 10^−2^	5.76 × 10^−2^	5.49 × 10^−2^	5.13 × 10^−2^	4.89 × 10^−2^	4.08 × 10^−2^
CO	Temporary work	8.68 × 10^−3^	8.68 × 10^−3^	8.51 × 10^−3^	7.90 × 10^−3^	7.73 × 10^−3^	7.62 × 10^−3^	6.84 × 10^−3^	6.84 × 10^−3^	6.67 × 10^−3^	6.67 × 10^−3^	5.41 × 10^−3^
	Structural work	3.45 × 10^−1^	3.40 × 10^−1^	3.33 × 10^−1^	3.26 × 10^−1^	3.19 × 10^−1^	3.13 × 10^−1^	3.04 × 10^−1^	2.99 × 10^−1^	2.92 × 10^−1^	2.87 × 10^−1^	2.74 × 10^−1^
	Finishing work	1.89 × 10^−1^	1.86 × 10^−1^	1.80 × 10^−1^	1.72 × 10^−1^	1.66 × 10^−1^	1.59 × 10^−1^	1.48 × 10^−1^	1.45 × 10^−1^	1.39 × 10^−1^	1.36 × 10^−1^	1.17 × 10^−3^
	Total	5.43 × 10^−1^	5.35 × 10^−1^	5.22 × 10^−1^	5.06 × 10^−1^	4.92 × 10^−1^	4.80 × 10^−1^	4.59 × 10^−1^	4.50 × 10^−1^	4.37 × 10^−1^	4.30 × 10^−1^	3.97 × 10^−1^
